# Design and Intensive Experimental Evaluation of an Enhanced Visible Light Communication System for Automotive Applications

**DOI:** 10.3390/s20113190

**Published:** 2020-06-04

**Authors:** Sebastian-Andrei Avătămăniței, Alin-Mihai Căilean, Adrian Done, Mihai Dimian, Valentin Popa, Marius Prelipceanu

**Affiliations:** 1 Integrated Center for Research, Development and Innovation in Advanced Materials, Nanotechnologies, and Distributed Systems for Fabrication and Control, Stefan cel Mare University of Suceava, 720229 Suceava, Romania; sebastian.avatamanitei@usm.ro (S.-A.A.); adone@eed.usv.ro (A.D.); dimian@usm.ro (M.D.); mprelipceanu@eed.usv.ro (M.P.); 2 Department of Computers, Electronics and Automation, Stefan cel Mare University of Suceava, 720229 Suceava, Romania; valentin@eed.usv.ro

**Keywords:** accident prevention, automotive applications, intelligent lighting systems, optical noise, vehicle safety, vehicular communications, visible light communication

## Abstract

As the interest toward communication-based vehicle safety applications is increasing, the development of secure wireless communication techniques has become an important research area. In this context, the article addresses issues that are related to the use of the visible light communication (VLC) technology in vehicular applications. Thus, it provides an extensive presentation concerning the main challenges and issues that are associated to vehicular VLC applications and of some of the existing VLC solutions. Moreover, the article presents the aspects related to the design and intensive experimental evaluation of a new automotive VLC system. The experimental evaluation performed in indoor and outdoor conditions shows that the proposed system can achieve communication distances up to 50 m and bit error ratio (BER) lower than 10^−6^, while being exposed to optical and weather perturbations. This article provides important evidence concerning the snowfall effect on middle to long range outdoor VLC, as the proposed VLC system was also evaluated in snowfall conditions. Accordingly, the experimental evaluation showed that snowfall and heavy gust could increase bit error rate by up to 10,000 times. Even so, this article provides encouraging evidence that VLC systems will soon be able to reliably support V2X communications.

## 1. Introduction

In the context in which the demand for wireless communication technologies is exponentially increasing in almost every domain [1,2,3], the 300 GHz radio-frequency (RF) and Microwave bandwidth becomes insufficient [4]. Thus, the usage of optical wireless communications (OWC) technologies [5] has become a promising alternative, as it provides almost unlimited bandwidth (i.e., more than 2000 times greater than RF and Microwave). Among OWC technologies, visible light communications (VLC) are of special interest from various points of view, providing the simultaneous usage of the visible light (380–780 nm) for illumination and communication purposes [6,7]. This dual use provides a significant energy efficiency, which is also improved by the use of solid state lighting sources providing cost-effective lighting and fast switching performances [8]. Moreover, the implementation cost of this communication technology is also reduced, because, unlike the RF and Microwave spectrum, the visible light spectrum is unregulated and, thus, communication licensing costs are eliminated. In addition to these technical advantages, a significant quality of VLC is that, unlike other communication technologies [9,10], this one is completely safe for human health.

**Figure 1:**
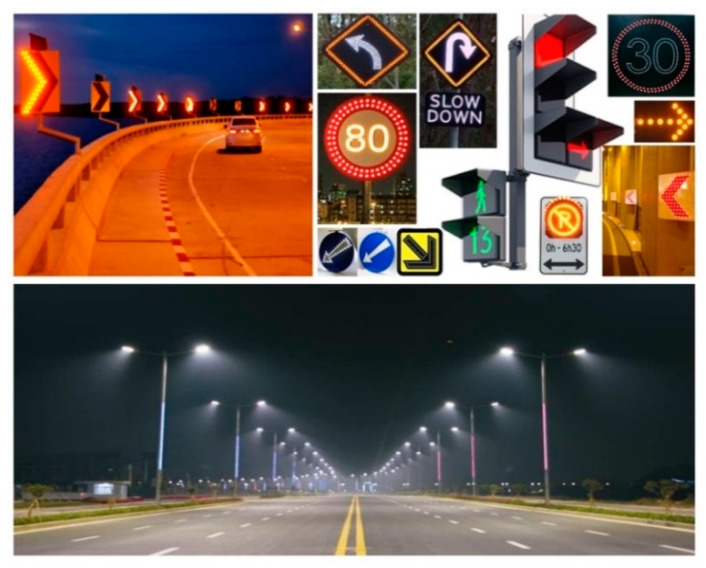
LED light sources integration in the transportation domain as part of vehicle lighting sources and of the transportation infrastructure. The wide distribution facilitates the usage of the visible light communications (VLC) technology.

Based on the upper-mentioned advantages, VLC technology is suitable for numerous applications. Thus, indoor VLC usage focuses on high data rate applications [11,12,13] and indoor localization [14,15]. On the other hand, outdoor VLC are strongly orientated toward automotive applications, including here communication-based vehicle safety applications [16,17,18] and inter-vehicle distance determination [19,20]. In these last cases, the use of the VLC technology in automotive applications is strongly favored by the wide distribution of LED lighting sources as part of vehicle lighting systems and transportation infrastructure, as illustrated in Figure 1. In this context, the development of automotive VLC systems emerged as a hot research topic addressing infrastructure to vehicle (I2 V) [16,21,22] and vehicle to vehicle (V2V) [18,23,24] applications.

Regarding vehicular applications, the existing RF-based communication solutions [25,26] perform well, but they are less reliable in a high vehicle density context [27,28]. In this situation, VLC-based systems could be used as a standalone solution or as a complementary technology for improved reliability and RF spectrum offloading [17,27]. The use of VLC in vehicular applications is favored by the specificity of the communications. Unlike RF-based communications, VLC are less affected by problems, like channel congestion, mutual interferences, or multipath. Consequently, the VLC technology could support the requirements that are imposed for vehicular communications in terms of high packet delivery ratio and reduced latencies [29,30]. VLC are a direct line of sight technology, which means that the problems generated by channel congestion and mutual interferences are mitigated by the spatial isolation between the neighboring VLC channels.

The VLC technology is currently considered to have high potential for vehicular applications; however, some are rather reserved regarding its immediate applicability due to existing challenges. An important step in confirming the potential of VLC technology in automotive applications is found in [18]. In this paper, the authors present the conclusions regarding the experimental testing of a V2V VLC link. This system has been tested in the real-world driving scenario and the results showed that VLC can provide almost continuous connectivity, even in mobile conditions and at highway speeds. These tests also indicated that the distance is the main factor affecting the link performances, again pointing out that middle to long range automotive VLC applications remain a challenge in the real-life use case.

Within this context, this article presents a new VLC communication system that is designed for communication-based vehicle safety applications. The proposed design is an enhanced version of VLC system that is able to achieve performances that further demonstrate the viability of VLC-based solutions in automotive applications. The transimpedance circuit is one of the most important elements of a VLC receiver, as it determines the resilience to optical noise sources. In almost every case, VLC systems use the linear transimpedance circuit. Nevertheless, it has been demonstrated that a logarithmic transimpedence circuit provides improved resilience to optical noise and an extended working range [31]. However, other factors must also be addressed in order to be fully compatible to automotive VLC systems. Thus, this article presents an enhanced VLC design by providing a VLC receiver solution capable to adapt the communication parameters to the specific context. Thus, unlike in [22,31], this new design is able to communicate while using different modulation techniques, coding techniques, and data rates (3–50 kb/s). Additionally, the novelty of this article comes from a more realistic approach on addressing the design of automotive VLC systems. In most of the existing works, custom made VLC emitters are used to demonstrate the potential of the technology [21,32], although they do not comply with the lighting requirements. Thus, in the real-world scenario, comparable results are rather difficult to be reproduced. Regarding the VLC sensors, most of the existing works report performances achieved under narrow field of view (FOV) conditions [16,32,33]. Although a narrow FOV VLC sensor is fully justified, as this approach significantly reduces the amount of parasitic light incident on the photosensitive element, the mobility of the system is reduced (especially in short range conditions). As the vehicular environment is defined as highly dynamic, such VLC systems are less compliant to the mobility requirements [17]. The proposed VLC emitter is developed using a standard compliant LED traffic light to address these issues. Thus, the results achieved by the system are like the ones obtained after the implementation of the concept in a real intersection and not overestimated by an increase of the emitted power. Furthermore, a wide angle FOV sensor has been used to enhance the system’s mobility and to maximize the compatibility with automotive applications instead of using a narrow angle VLC sensor to minimize the effect of parasitic light. Of course, these two approaches significantly decrease the signal to noise ratio (SNR) and complicate the data reconstructing process. The proposed system was tested under different working scenarios. The experimental testing has confirmed the system’s performances, its ability to work under noisy conditions, and its capacity to achieve communications distances up to 50 m. Thus, the proposed design represents a simple, low-cost, and easy to implement solution for a safer road network. Another aspect that is addressed in this article is related to the modulation and coding techniques that are suitable in vehicular VLC applications. Hence, this work investigates the performances of a spread spectrum based modulation suitable for poor SNR scenarios or for high priority data, the performances of a classical modulation and coding technique suitable for general purpose applications/scenarios, and the performances of a third coding technique that could be more appropriate in multiple input multiple output (MIMO) applications in order to path the way toward context adaptive VLC systems [17,34]. All of these techniques are investigated through intensive experimental evaluation performed in laboratory conditions and in outdoor uncontrolled conditions. Consequently, another important contribution of this article consists of a decisive step toward the design, development, and implementation of context adaptive VLC architectures compatible to the strict requirements imposed for automotive applications. Finally, another important contribution of this work comes from the fact that it provides one of the very few experimental evaluations of an automotive VLC system in middle- to long-range outdoor snowfall conditions.

The rest of this article is organized, as follows. Section 2 approaches the issues that are related to the use of the VLC technology in vehicular applications, pointing out some of the challenges and presenting some of the best existing VLC solutions. Section 3 presents the proposed VLC system in terms of hardware and software components. Section 4 details the intensive experimental verification of the proposed VLC solution. Section 5 provides a discussion concerning the experimental results and the ability of VLC systems to comply with automotive applications, whereas Section 6 provides the final conclusions of this article.

## 2. Vehicular Visible Light Communication Issues, Related Work, and Specificity of this Work

### 2.1. Automotive VLC Specific Issues

Visible light communications in general and automotive VLC applications, in particular, are a highly complex research areas, as numerous issues influence the communication performance, especially in outdoor conditions. In the most popular approach, the VLC sensor mainly consists of a photosensitive element, usually, a reversed bias positive intrinsic negative (PIN) photodiode, connected in a transimpedace circuit [31]. The photodiode generates an electrical current that is directly proportional to the incident light. Thus, significant challenges already begin to arise:

i. as the generated photocurrent is directly proportional to the incident (i.e., data signal carrying light), whereas, as the ***optical power is decreasing proportionally to the distance’ square***, middle to long-range automotive VLC applications must cope with ***very low power optical signals*** that can go down to tens of nW/cm^2^ levels;
ii. unlike other wireless communication technologies, in VLC the ***optical carrier is perceivable to the human eye***, and so, the ***optical transmission power is strictly determined by the lighting device primary purpose*** and by light regulations, with no possibility to increase it;
iii. automotive VLC applications must cope with a ***multitude of weatherphenomena that influence the light passage***; thus, heavy dust can block part of the light [35], fog and rain influence the light passage by means of reflection, refraction, absorption, and scattering [36,37,38], whereas sleet and snow also affect the light passage affecting the received optical power [39,40]. Hence, weather phenomena block part of the light affecting, in turn, the SNR and the communication range. The effect of the snow is probably the most disruptive on automotive VLC, as dense snowfall can reduce the VLC communication distance by up to 60–80%, as demonstrated by simulation means [40];
iv. in order to have connectivity between the VLC transmitter and the VLC receiver, the emitted light must reach the photosensitive surface and, thus, the ***line-of-sight condition is mandatory*** and affects the mobility and the connectivity of the automotive VLC link. In order to address this issue the complexity of the system design must be increased [17,41]. A relay assisted approach to address this issue is under research consideration [42,43]; and,
v. as the photodiode generates an electrical current proportional to all the incident light, one of the main problems that emerge is generated by the ***multitude of light sources*** that are ***present in the outdoor environment***. Hence, the photodiode will be exposed to very strong sunlight and artificial light sources (i.e., incandescent, fluorescent, LED). Most likely, the power of the parasitic light is considerably higher than the power of the data carrier light (i.e., thousands of times higher) significantly affecting the SNR. Thus, the effect of parasitic light sources is the most important problem affecting the performances of automotive VLC. Nevertheless, these aspects are not detailed in this work again, as this topic has been widely debated [16,17,18,31,44,45], whereas additional information concerning optical noise modeling and its influence on the VLC system can be found in [46,47,48].

One can see that numerous issues in the outdoor environment affect automotive VLC applications. Most of these perturbing elements affect the light passage and introduce additional noise to the system, significantly affecting the SNR. Thus, in the real use case scenario, automotive VLC applications must cope with low SNR signals while providing a highly reliable link.

### 2.2. Research Trends in Automotive VLC Applications

Recent years have shown important progress in the area of automotive VLC applications. Still, there are few steps ahead before having a VLC system that is fully compatible with automotive applications. Simulation results [49,50,51,52] indicate that VLC systems could be capable of fulfilling these requirements, whereas, in favorable cases, such systems could provide 50 Mb/s data rates and communication ranges of up to 70 m [53]. However, existing systems are not able to experimentally demonstrate such performances so far. Thus, several challenges still need to be addressed. Accordingly, from the physical point of view, automotive VLC systems must be able to further enhance their (i) resilience to ambient light sources, (ii) communication range, (iii) data rate, and (iv) ensure their compatibility to the mobility of vehicular applications [17]. Nevertheless, high performances on a specific feature will not be able to provide a ready-to-deployment vehicular VLC system. Hence, future automotive VLC systems must be able to ensure decent overall performances, while achieving an optimal tradeoff between different characteristics (i.e., noise resilience, communication distance, mobility, and data rate). This could be achieved by developing environment and context adaptive VLC systems [34]. This concept envisions that the system is analyzing the specific context and adjusts the configuration in order to maximize the performances and achieve the most optimal trade-off between the different parameters.

### 2.3. State of the Art Summary and the Position of this Work with Respect to Other Works

Although important progress has been achieved, there are limited experimental demonstrations that show a VLC system that is able to fully comply with the requirement of the automotive area. Thus, most existing systems can have remarkable performances, as they focus on a specific challenge. However, there is limited work focused on a holistic approach that leads to a VLC system that is capable of providing mobility, long-range, high data rates and resilience to optical noise sources and extreme weather phenomena. Thus, significant work in this area is still required. In this context, this work aims to provide a VLC system that is designed based on a holistic approach that focuses on the overall system performances. The proposed prototype is designed to work in variable scenarios, distances, under different optical noise exposure levels, in unfriendly meteorological conditions, and so on. Thus, unlike many of the existing VLC prototypes, this one is not tested only in ideal conditions or in circumstances that emphasize a specific ability (i.e., noise resilience, data rate, communication range), but it is also evaluated in a multitude of situations, favorable and unfavorable. Accordingly, when compared to the VLC systems and solutions that are focused on noise mitigation, the one that is presented in this work aims to provide similar resilience to optical noise sources (i.e., up to few thousands of lux), while being able to provide a superior communication range than [21,23], better mobility than [33], and a simpler design than [54,55,56,57]. When compared to the vehicular VLC systems that address tens of meters communication ranges, the proposed prototype aims to provide higher resilience to noise than [16,21,30,58,59], wider FOV and better mobility than [16,58,59], and lower complexity than [60]. On the downside, the proposed VLC prototype achieves significantly lower data rates than [59,61,62,63,64,65,66,67], but it provides better resilience to noise and longer communication ranges ([64] provides a rather similar communication range). Thus, high-speed camera systems that are based VLC systems can achieve data rates up to few tens of Mb/s data rates [61,62,63], whereas some of the short-range (i.e., 1–3 m) photodiode-based vehicular VLC systems can provide hundreds of Mb/s data rates [60,65,66,67]. Additionally, as compared to most of the upper-mentioned VLC systems, this one is tested in a wider range of scenarios (i.e., indoor and outdoor, short range to long range, different optical noise intensities, unfriendly weather conditions) in order to demonstrate the benefits of a holistic approach in vehicular VLC design.

## 3. Description of the Proposed VLC Prototype

### 3.1. Hardware Design and Implementation

**Figure 2:**
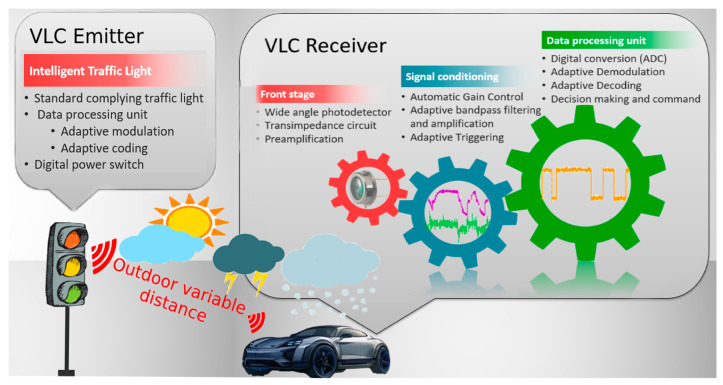
Diagram of the visible light communications system. The VLC receiver encloses a logarithmic transimpedance circuit that is able to receive signals at variable power intensities, adaptive gain, adaptive filters, adaptive triggering, and adaptive decoding. These functions enable it to have an optimal configuration adapted to the changeable atmospheric conditions.

In the upper-mentioned context, this article proposes a new VLC system that is designed for traffic safety applications. Although the functionality of the system in automotive applications is illustrated using a traffic light as a VLC emitter, the module transforming the light source into a VLC emitter is compatible with any LED lighting or signaling device that can be found in the transportation system. Figure 2 illustrates the schematic of the proposed system. The system consists of a VLC emitter and a VLC receiver that is designed to be compatible with the automotive domain.

#### 3.1.1. Discussions on the Design of the VLC Emitter

In order to demonstrate that any LED-based traffic light can be updated in order to become an intelligent data broadcasting device, the VLC emitter is developed based on a standard 200 mm LED-based traffic light. The commercial traffic is in agreement with the SR EN 12368 [68] and the SR EN 60598-1 [69] standards and its emitted light has an irradiance of 190 μ/cm^2^ (i.e., measured at 1 m).

**Figure 3:**
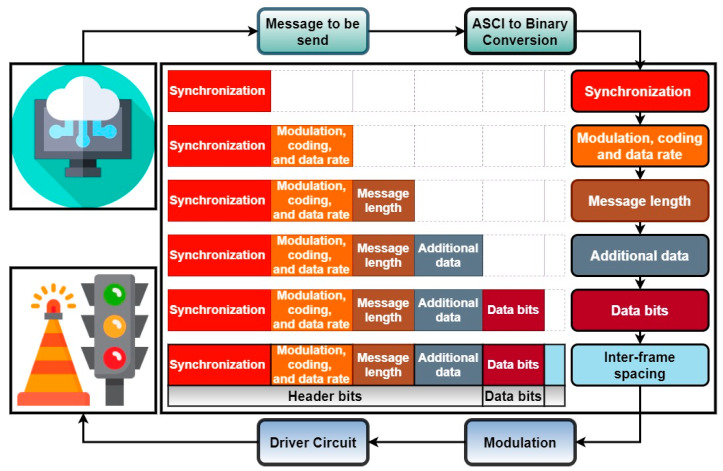
Schematic of the frame building at the VLC receiver level.

This is a very important aspect, because, in other works, custom made VLC emitters having different characteristics are used. Usually, these custom made traffic lights have powers that are significantly higher than the ones that are specified in the traffic equipment standards [32]; they are using a significantly higher number of LEDs to enable MIMO communications with camera-based VLC receivers [60] or they have a modified light radiation pattern. All of these modifications enable such systems to achieve enhanced performances in terms of communication distances, data rates, and bit error ratio (BER). Although, for technology demonstration purposes, these approaches are fully justified, as they provide a hint on the achievable performances, the usage of standard complying equipment should be investigated, as it provides a realistic view on the performances in real situations. Thus, using commercial traffic lights that are similar to the ones that are found in every intersection and that follow the requirements of the existing standards provides an important step in confirming the compatibility between the VLC technology and automotive applications. The LED traffic light has been enhanced with an external module consisting of a data processing unit and a digital power switch. A 180 MHz microcontroller has been used for data processing due to cost and efficiency reasons. The microcontroller is the central element of the VLC emitter, as it is responsible for message generating, coding, modulating, and frame building. Based on the data to transmit sequence, the microcontroller commands the switching of the LEDs through the digital power switch. Accordingly, an amplitude-modulated light beam is generated and transmitted through the free space optical channel, as illustrated in Figure 3.

#### 3.1.2. Discussions on the Design of the VLC Receiver

A VLC receiver architecture is proposed to achieve the objective of providing a superior VLC system. The VLC sensor is developed while considering its future integration into commercial vehicles and, thus, the cost constraints were considered to be a fundamental aspect. Furthermore, the development of each functional block has been made while considering the challenging outdoor scenario and the requirements of vehicular communication applications. Hence, each functional block of the sensor is specially designed to be able to cope with a multitude of scenarios and conditions.

The VLC sensor uses a PIN photodiode working in the photoconductive mode and coupled in a transimpedance circuit. It is well known that avalanche photodiodes (APD) provide improved sensitivity when compared to PIN photodiodes. Nevertheless, the SNR is usually affected, as they also amplify the noise. Based on the fact that this system is intended to work in variable uncontrolled outdoor conditions, the use of a PIN photodiode is preferred. This decision can reduce the communication range in night circumstances, but it significantly improves the SNR in daytime conditions. Because the system is envisioned to provide a holistic approach, focusing on the overall system performances, a PIN photodiode seems to be a better choice. The photodiode generates an electrical current proportional to the incident light. The transimpedance outputs a proportional voltage that is further processed until the data are recovered. Selecting the sensor’s FOV represents an important decision, as VLC are a direct line of sight technology. A narrow FOV reduces the influence of the parasitic light on the system performances [33], but it limits the mobility [17]. Because the VLC sensor is envisioned for the automotive domain, whereas vehicular communications involve continuous mobility, a wide angle FOV of ±45° was considered. Consequently, in daylight conditions, the parasitic light will influence the generated photocurrent, while the data extracting process becomes more challenging. In order to reduce this influence, the receiver’s bandwidth is limited to 300 kHz, while a logarithmic transimpedance configuration [31] is used instead of the classical one.

Next, two amplification stages amplify the output of the transimpedance circuit. However, as vehicular communications involve high dynamicity, the power of the received signal can vary significantly, as the emitter–receiver distance is changing. Moreover, in high background brightness conditions, the sensitivity of the logarithmic transimpedance circuit is automatically adjusted in order to prevent photoelement saturation, further limiting the amplitude of the useful signal, as mentioned above. An additional automatic gain control (AGC) stage is used to provide a constant amplitude signal to address these issues. This solution is able to improve the BER results within the entire communication service area. As the sensor is envisioned to receive data modulated at variable frequencies between few kHz and few hundreds of kHz (i.e., although the bandwidth is currently limited to 300 kHz, higher frequencies are envisioned for low background light conditions), a specific condition that was imposed for the AGC stage was to provide a constant signal gain, whatever the frequency of the data (i.e., for frequencies within the interest band).

Subsequently, the signal passes through a filtering stage that limits the passage of low and high frequency noise. As the sensor is envisioned to work with changeable frequencies, this block is designed to be able to commute between several frequency bands. The high pass filter is set at 1 kHz cutoff frequency and it has the purpose of removing low frequency interferences mainly introduced by incandescent and fluorescent lighting sources. The adaptive filter also consists of several selectable 4th order Bessel low pass analogic filters that were obtained using a Sallen-Key configuration. In this case, the number of modulation frequencies in use determines the number of parallel filters (i.e., currently the VLC system works with three modulation frequencies). The Bessel filters have been considered due to their ability to prevent significant signal phase distortions within the bandpass. The cutoff frequency for the low-pass filters is individually established for each communication frequency. The microcontroller unit controls the selection of the active frequency band, which is able to make this decision based on the information extracted from the message header. Basically, the synchronization header of each message provides the VLC receiver with information regarding the frequency of the incoming data. Based on this fact and when considering the power spectral density distribution of the encoded data, the microcontroller selects the optimal configuration of the adaptive filters (i.e., the filter having the most appropriate output signal). In the current configuration, the VLC receiver is able to process the data transmitted using frequencies between 22–100 kHz, whereas frequencies up to 1 MHz are envisioned.

In the next phase, the output of the filtering block is introduced in a Schmitt trigger circuit providing the digital data signal. The reconstructed signal is processed by the 180 MHz microcontroller, which extracts the data after demodulation and decoding. The signal is analyzed based on a pulse width measurement algorithm in order to decode the data. This implies the identification of the rising and of the falling edges as well as a time measurement between these two events. The microcontroller is able to decode messages transmitted using direct sequence spread spectrum (DSSS) modulation along with sequence inverse keying (SIK) coding, and on-off keying (OOK) modulated messages while using Manchester, respectively, Miller coding (delay modulation). Nevertheless, the microcontroller must know which decoding algorithm to use in order to properly extract the data. Hence, the information regarding the configuration of the data (i.e., modulation, coding technique, frequency, message length) is also provided in the message header (see Figure 3). Founded on this flexible configuration, the VLC receiver should be able to operate under both ideal and unfavorable conditions.

### 3.2. Software Design and Implementation: Discussions on the Modulation Techniques, Coding Techniques, Data Rates and Communication Protocols

#### 3.2.1. Modulation and Coding Techniques

Outdoor low data rate applications should use OOK modulation and Manchester coding, achieving data rates between 11 and 100 kb/s, according to the IEEE 802.15.7 standard for short wireless optical communications using visible light [7]. The performances of OOK and of Manchester coding are well established in the literature. Manchester’s code advantages are given by its DC balanced characteristics, simple clock and data recovery, and by the decent BER performances. Furthermore, in VLC applications, the Manchester code is suitable due to its ability to prevent light flickering. In the Manchester code, a binary 1 is represented as a high to low mid-bit transition, whereas a binary 0 is represented as a low to high mid-bit transition. Thus, while transmitting a Manchester encoded message, the light of each data bit is constant, which prevents any intra-frame flickering. On the down-side, the Manchester code has the disadvantage of larger bandwidth requirements as compared to other common codes. Accordingly, in addition to the classical Manchester code, the performances of the Miller code [70,71] are also investigated. The Miller code has rather similar advantages as the Manchester code, but it is also bandwidth-efficient. Consequently, the use of the delay modulation could be a solution that is suitable for MIMO applications. This characteristic is given by the fact that the energy of the Miller encoded data is concentrated at a lower frequency and within a narrower bandwidth. Therefore, bandpass filters become more efficient in separating adjacent channels.

DSSS modulation with SIK coding is the second modulation technique under consideration. DSSS is a robust modulation technique that uses spread spectrum and sequence inverse keying [21,72]. This modulation increases the communication robustness to noise and to potential multipath dispersion at the cost of a much larger bandwidth requirement. Basically, the information is expanded on a much wider spectrum, increasing the robustness to light interferences. In this case, the data are transmitted using sequence inverse keying, which means that a binary 1 is represented as a pseudo-noise sequence of zeros and ones, whereas binary 0 is represented by its complement. The length of the pseudo-noise sequence is established as a compromise between robustness to noise and bandwidth. In this specific case, a 7-bit noise sequence has been selected. Thus, a binary 1 is represented as 1010101, whereas a binary 0 is represented as 0101010. In such a case, the information can still be extracted when up to three bits of the pseudo noise sequence are affected by noise.

In conclusion, the prototype is designed to work with two types of modulations and three types of coding techniques. The OOK modulation along with Manchester coding represents the standard solution for such applications, whereas the Miller code is considered for its improved bandwidth efficiency and its potential use in MIMO applications. Additionally, for the case of low SNR conditions, DSSS modulation along with SIK coding is considered. In this last case, the data rate is sacrificed in order to maintain the link active.

#### 3.2.2. Data Rates and Communication Protocols

Unlike indoor VLC applications where multi-Gb/s data rates are achievable due to the short distances involved and, due to the low noise conditions, outdoor applications in general and vehicular applications in particular envision significantly lower data rates. For example, the VLC system presented in [60] is able to achieve data rates up to 20 Mb/s at short-range, whereas, as the distance is increasing up to 110 m, the data rate is decreasing at 1.5 kb/s. Thus, in vehicular communication applications, the link robustness is more important than the data rate, as a lower data rate is preferable instead of a communication breakdown [17].

Based on this consideration, the proposed prototype is designed to work at variable data rates within the 3–50 kb/s range. Thus, for medium to low noise conditions, the use of OOK modulation is considered. For low SNR conditions and extreme working conditions (i.e., fog, snow, direct sunlight exposure), the system is designed to use DSSS modulation at a frequency of 22 kHz and, thus, a 3 kb/s data rate is achieved. Although, in this case, the achievable data rate is quite low, the use of DSSS modulation is envisioned for scenarios in which the SNR is very low or when the noise frequency is within the VLC receiver bandwidth.

When considering the imposed requirements (i.e., modulation, coding, data rate), the VLC emitter transforms the data to send into an amplitude modulated light beam. Thus, the text message data are converted into a binary string, which is then encoded, modulated, and transmitted over the free space optical channel (as illustrated in Figure 3). For simplicity and cost constraints reasons, the system uses asynchronous transmission, with the synchronization information being located in the message header. The header also contains information regarding the modulation, the coding, the frequency, and the message length. The VLC receiver uses all of these data in the data recovery process.

## 4. Experimental Evaluation of the VLC System and Experimental Results

**Figure 4:**
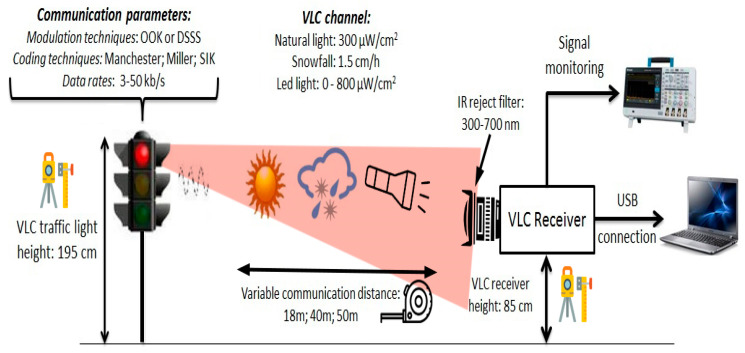
Schematic of the experimental setup.

The following section presents the experimental evaluation of the proposed VLC system. The purpose of these tests is to confirm the functionality and viability of the system under different conditions and testing scenarios. Thus, the performances of the VLC system are investigated in different noise conditions and for variable communication distances. Moreover, the evaluation process also examines the performances of the system for different modulation techniques, coding techniques, and communication frequencies. Thus, this experimental verification investigates the potential benefits that are associated with context adaptive VLC architectures (i.e., the communication parameters are selected based on the existing context). Furthermore, the aim of the experimental verification is to confirm the compatibility between VLC and automotive applications. This objective is motivated by the fact that the proposed system is developed while using a commercial standard compliant LED traffic light and a wide FOV VLC sensor. Last but not least, these tests are also intended to confirm the benefits of the logarithmic transimpedance circuit. The experimental setup is illustrated in Figure 4, whereas its summary can be found in Table 1. Additional details concerning each test are presented within the dedicated section.

### 4.1. Experimental Setup No. 1—Medium Communication Distance in High SNR Conditions

The aim of the next set of experiments was to evaluate the functionality of the system in low noise conditions and to also evaluate the performances of the two modulation techniques. In the first experimental setup, the VLC emitter and the VLC sensor were placed 18 m away from each other within a corridor of the laboratory. The power of the emitted light measured at 1 m is 190 μW/cm^2^. As any electromagnetic radiation, the power of the transmitted light decreases exponentially as the distance is increasing. Furthermore, the power reaching the receiver is also influenced by the angle between emitter and receiver. Thus, the measured power for the received signal is 0.56 μW/cm^2^, whereas the power of the natural light is between 100–300 μW/cm^2^.

Data sets of about 10 million-bit have been transmitted by repeating a predefined 72-bit text message in order to determine the BER. After reconstructing the data signal (analog signal processing), it is then transformed into a digital message that is processed by the 180 MHz microcontroller. The microcontroller generates an interruption on every rising and falling edge of the digital signal. Thus, the difference between two consecutive interruptions determines the pulse width. Based on this width, and by knowing the code used in the message coding (this info is extracted from the message header), the microcontroller is able to establish the bit value (i.e., 1 or 0). This value is stored in the microcontroller memory and it is than compared to the bits that it supposed to receive. After each comparison, a counter is incremented for correct or incorrect bits. The microcontroller does not use the serial connection while it is processes the data in order not to perturb the data decoding process and enable real-time BER processing. The data have been transmitted using DSSS modulation and SIK coding, OOK modulation and Manchester coding and OOK modulation, and Miller coding. For the data that are transmitted using DSSS, a modulation frequency of 22 kHz has been selected. For OOK modulation, the data have been transmitted using three different data rates 11 kb/s, 24 kb/s, and 50 kb/s. The VLC emitter was placed at a height of 190 cm, whereas the VLC receiver at a height of 85 cm. Table 2 presents the summary of the experimental results. The experimental results confirm the suitability of the proposed design. Thus, for both modulation techniques BERs lower than 10^−6^ have been achieved. These BER results have been determined for the physical layer, without using any error correcting methods. 

### 4.2. Experimental Setup No. 2—Medium Communication Distance in Low SNR Conditions

**Figure 5:**
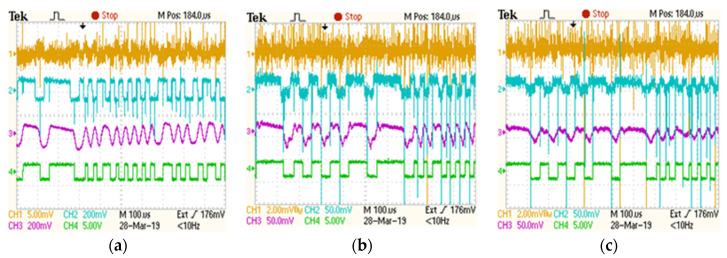
Oscilloscope screens illustrating the signal reconstruction process at the level of the different blocks of the VLC sensor: Channel 1 (orange) shows the output of the logarithmic transimpedance circuit; Channel 2 (blue) shows the output of the transimpedance circuit after a 24× amplification; Channel 3 (purple) shows the output of the filtering block; Channel 4 (green) shows the reconstructed data signal. The figure shows the response of the VLC receiver as the power of the incident parasitic light is increasing: (**a**). diffuse natural light having a power of 150 μW/cm^2^; (**b**). direct LED light having a power of 400 μW/cm^2^; (**c**). direct LED light having a power of 800 μW/cm^2^. One can see the response of the VLC receiver: as the SNR deteriorates the logarithmic transimpedance circuit reduces its sensibility in order to prevent the photoelement saturation.

The purpose of the following experimental setup is to investigate the influence of parasitic noise sources on the communication’s performances. Another important aim for these tests was to confirm the benefits that are associated with the use of a logarithmic transimpedance circuit instead of the classical approach. For this setup, the emitter-receiver distance is maintained at 18 m, while a perturbing high power LED has been placed 17 cm from the receiver. The VLC sensor receives an additional direct optical noise of 190 μW/cm^2^ due to the perturbing LED. Even though the noise source is directly facing the VLC receiver, whereas the received signal power is significantly lower, the VLC receiver is still able to extract the data. Figure 5 demonstrates the signal reconstruction process within the main stages of the VLC receiver. It illustrates the output of the logarithmic transimpedance circuit, the output of the first pre-amplification block, the output of the filters, and the reconstructed data signal. This figure also shows the sensor’s response to the noise power increase (Figure 5b,c). One can see that, as the level of the received perturbing light is increasing, the logarithmic transimpedance circuit is counteracting this effect by automatically adjusting its response. Thus, the photoelement saturation is prevented. The logarithmic transimpedance circuit response to mitigate the noise effect also leads to a decrease in the data signal amplitude. Nevertheless, the proper response of the AGC circuit compensates the above mentioned issue, whereas the reconstruction of the data signal is not affected. Table 3 presents the summary of these experiments. The experimental results showed that, even when a moderate noise source is perturbing the VLC receiver, it is still able to maintain the BER lower than 10^−6^.

### 4.3. Experimental Setup No. 3—Long Communication Distance in Low SNR Conditions

**Figure 6:**
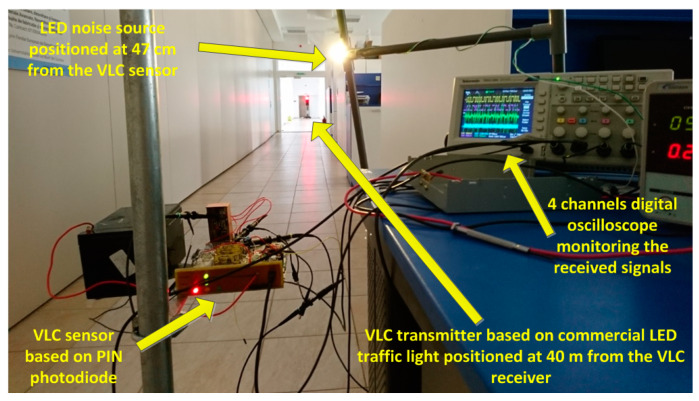
Picture of the experimental setup with the VLC emitter placed 40 m away from the VLC receiver, a LED noise source placed at 47 cm from the VLC sensor, and the oscilloscope showing the received signal.

**Figure 7:**
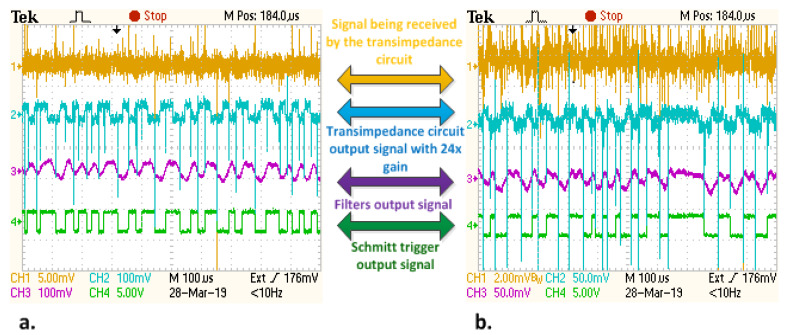
Oscilloscope screens illustrating the signal reconstruction process at the level of the VLC receiver for a 40 m communication distance: (**a**) in normal conditions (**b**) with white LED perturbing optical noise source. One can see the effect of the perturbing noise source on the data signal and the response of the logarithmic transimpedance circuit.

The purpose of the next experimental setup is to evaluate the behavior of the proposed system at longer communication distances. In this case, the communication distance was increased up to 40 m and the behavior of the communications has been analyzed for the two modulation techniques. At this distance, the power of the received signal is 0.11 μW/cm^2^. The picture of the experimental setup is available in Figure 6. Figure 7a illustrates the signal reconstruction process within the receiver at the level of the different signal processing blocks for this distance. The SNR level was further reduced, as the VLC receiver is still able to properly reconstruct the data signal. Thus, a perturbing white LED was placed at 47cm toward the VLC receiver. In these conditions, the power of the parasitic LED light measured at the sensor level was 120 μW/cm^2^ (in addition to the power of the natural light—see Table 1). The effect of the parasitic light on the VLC receiver can be observed in Figure 7b. One can see that the logarithmic transimpedance circuit automatically degreases its gain in order to prevent the photosensitive element saturation. Thus, the amplitude of the voltage that is produced by the logarithmic transimpedance circuit becomes two to three times lower (Figure 7b). Furthermore, the strong perturbing light also affects the shape of the signal, as it can be observed at the output of the filtering block (Figure 7b—Channel 3). Hence, the signal loses its square shape, whereas the period of the data bits is slightly affected. Nevertheless, the data message can still be decoded, as the frequencies involved are quite low, whereas the tolerances are reasonably high. Table 4 presents the summary of the experimental results for this setup. The use of the DSSS modulation enables the system to maintain a BER lower than 10^−6^. This BER is also achieved for OOK modulation when data rates of 11 or 24 kb/s are selected. However, the BER decreased for both Manchester and Miller codes when the data rate was increased to 50 kb/s. In conclusion, in the current configuration, the 50 kb/s data rate should only be used for low priority data, in high SNR conditions or in conjunction with error correcting codes.

### 4.4. Experimental Setup No. 4—Long Communication Distance in Outdoor Snowfall Conditions

After presenting the experimental results in variable indoor conditions, the next step is to provide the experimental validation in outdoor surroundings. The logarithmic transimpedance circuit is able to significantly reduce the possibility of saturation, while expanding the sensor’s dynamic range (i.e., the system is able to work in a wide range of background lighting), as shown in [31]. Thus, as verified in [31], the logarithmic transimpedance circuit enables the VLC receiver to provide a 10^−6^–10^−3^ BER at 50 m, in sunny day conditions (i.e., sunlight irradiance up to 67000 µW/cm^2^).

In addition to strong sunlight, snowfall represents the most important perturbing source for outdoor VLC, as demonstrated in [40]. Snowfall can reduce the communication range by up to 60–80%, according to [40]. Consequently, the following test is evaluating the proposed VLC system performances in snowfall conditions for a 50 m communication range. In addition to distance and snowfall, the VLC channel is also affected by heavy blizzard episodes. The summary of the weather conditions during the experimental evaluation is available in Table 5, whereas a configuration of the VLC system during these tests is presented in Table 6.

**Figure 8:**
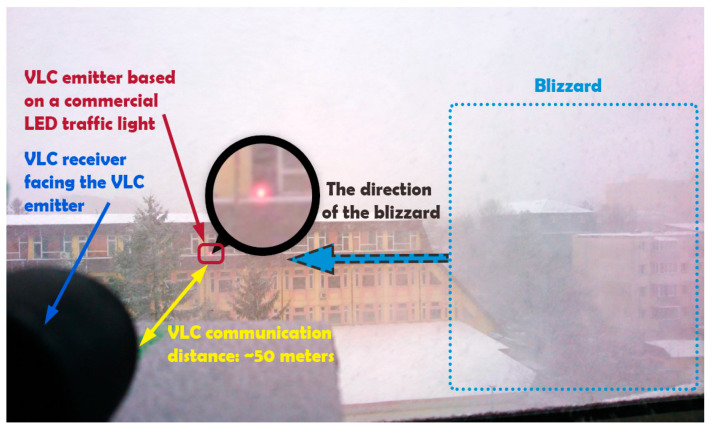
Experimental evaluation of the proposed vehicular VLC system in outdoor snowfall conditions for a 50 m VLC traffic light emitter to VLC receiver communication distance. In this setup, the VLC channel is perturbed by snowfall and heavy blizzard episodes.

**Figure 9:**
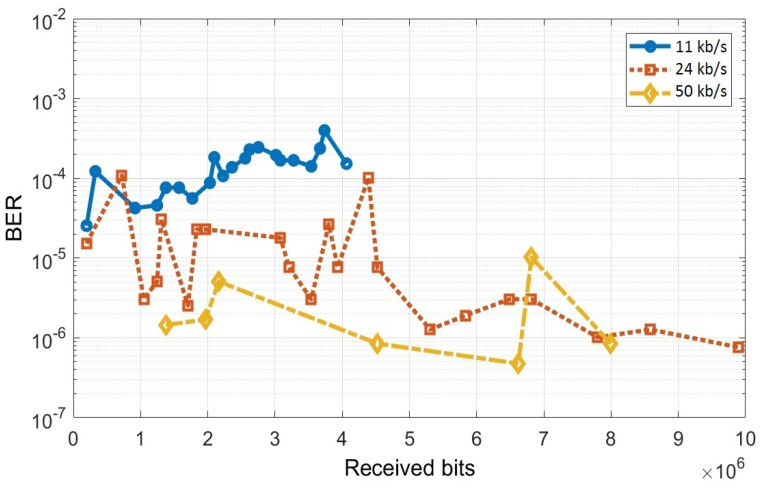
Real-time evolution of the BER results in snowfall and wind gust conditions. The peeks in the BER representation are generated by variations of snowfall intensity and by wind gusts. Additionally, as these tests were performed in outdoor uncontrolled conditions, the lower BER results illustrated for the higher data rates are due to a lower intensity of the snowfall.

Figure 8 shows the experimental setup, whereas Table 7 shows the summary of experimental results. These results showed that snowfall has the potential to increase the number of bit errors by 100 times, whereas the number of bit errors can increase by up to 10,000 times when the snowfall is also associated to heavy gust. Thus, the experimental evaluation has confirmed the simulations from [40], demonstrating the highly disruptive effect of snowfall and blizzard. Figure 9 shows the real-time evolution of the BER for three data rates while using OOK modulation and Manchester coding. This figure shows the manner in which the BER evolves and how it is influenced by the intensity of the snowfall or of the wind gusts.

One can also see from Figure 9 that, in this case, the experimental results show that the BER is decreasing as the data rate is increasing. Nevertheless, these results are influenced by the snowfall and the wind gust intensities, as the experiments have been made in uncontrolled snowing conditions. From these reasons, the DSSS modulation and the Miller code have not been evaluated in this experimental setup in order to prevent any misleading of the reader, as the results could have been ambiguous.

### 4.5. Communication Latency Evaluation

In addition to noise resilience, communication ranges, and data rates, wireless vehicular communications also impose strict requirements for communication latencies. Accordingly, a message transmitted by a vehicle must reach the destination vehicle within a certain time limit. As defined in [73] and summarized in [27], this limit is established as a function of the application. For example, a message containing a curve speed warning must reach its destination within a 1000 ms limit, whereas a message referring to a traffic signal violation must have its latency lower than 100 ms (latencies that are below 100 ms are imposed for the majority of high priority safety application messages). The strictest latency requirement is imposed for the scenario when two vehicles are on a collision trajectory. In such circumstances, in order to prevent accident occurrence, latencies below 20 ms must be provided. Nevertheless, existing research has shown that, in high traffic density scenarios, 5.9 GHz DSRC systems are not able to guarantee such requirements [27] and, so, the usage of the VLC technology as a complement technology can further improve the communication reliability.

**Figure 10:**
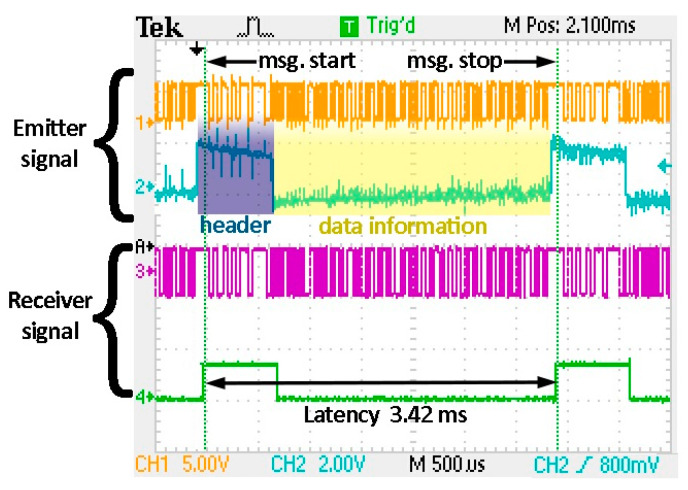
Communication latency measurement. For a clear presentation this example illustrates a 56 bit data message transmitted at 24 kb/s using on-off keying (OOK) modulation and Manchester coding. time and data processing time at the VLC receiver level. Channel 1 (orange) represents the message at the VLC emitter, Channel 2 (blue) shows the beginning of a message where the high pulse represents header transmission and the low pulse represents data message transmission, Channel 3 (purple) illustrates the received message, whereas Channel 4 (green) shows header processing.

In these circumstances, the next test is performed in order to evaluate the proposed VLC prototype in terms of latency performances. Similar as in [29], the latency of the system is considered to be equal to the time difference between the moment that the VLC emitter begins to transmit the data and the moment the VLC receiver decodes the entire message. Based on this definition, one can see that there are several factors influencing the latency. The main ones are given by the transmission data rate and by the length of the data message. According to [73], messages transmitted in vehicular communications high priority safety applications have a length between 208 and 904 bits, whereas a 400-bit message is conserved as an average message length. Accordingly, these tests were performed for a 400 bits message length. Figure 10 shows the latency measurement procedure. One can see here that the 180 MHz microcontroller is able to process the data in real time, whereas the electronic circuits introduce a negligible latency (i.e., below 200 µs). Table 8 summarizes the results of these tests. One can see that, when the VLC system is working at data rates of 24 kb/s or above, it is able to satisfy even the strictest latency requirements (i.e., below 20 ms) for a message of up to 400 data bits. For lower OOK data rates, the system is suitable for most applications with the exception of pre-crash sensing cooperative collision mitigation. On the other hand, these tests have showed that the usage of the 3 kb/s DSSS modulation should be used only for shorter messages to ensure latency below 100 ms. Overall, these experimental results confirm that the proposed VLC system is able to satisfy the latency requirements imposed in communication-based vehicle safety applications. Moreover, the system latencies will be further reduced, as these data rates will be further improved. Accordingly, from this point of view, VLC systems are definitely suitable for automotive applications.

## 5. Final Discussions Concerning the Proposed VLC Prototype

The proposed VLC system has been evaluated under variable scenarios, distances, and noise conditions in order to demonstrate the compatibility of VLC systems with vehicular communication applications. As this work involves the development of a context-adaptive VLC system, different modulation techniques, coding techniques, and data rates have also been investigated. Thus, the proposed VLC architecture aims to simultaneously address all of the main issues affecting vehicular VLC, while providing an optimal trade-off between different parameters of the system. Accordingly, in order to facilitate the deployment of automotive VLC systems, their future development should be focused on improving specific abilities (i.e., resilience to noise, communication range, data rate, mobility), but also on a holistic approach that addresses the overall performances. In this context, the proposed VLC design brings together in one system performances of state of the art VLC systems. Thus, it provides noise resilience that is comparable with [21,23,33,54,55,56,57], but longer communication distance than [21,23], better mobility performances [33], and simpler design than [54,55,56,57]; it ensures alike communication distances as [16,21,30,58,59], but improved noise resilience when compared with [16,21,30,58,59] and improved mobility as compared with [16,58,59]. On the downside, in the current configuration the proposed VLC prototype is able to provide 3–50 kb/s data rates. Although these data rates are comparable with the ones achieved by other middle-long range automotive VLC systems [16,18,19,20,30], they are lower when compared to the 5.9 GHz DSRC systems, which can achieve few Mb/s data rates. Thus, this aspect should be further improved in order to provide higher data rates as long as these advances do not affect noise resilience and link reliability.

The experimental verification has confirmed the high potential of VLC systems in vehicular applications. It should be remembered here that these tests have been made while using a standard VLC traffic light and a wide FOV VLC receiver (i.e., suitable in mobile applications). Thus, in such conditions, the SNR becomes significantly lower, complicating the signal reconstruction process. According to the existing standards, 200 mm traffic lights are fixed at 250 cm above the road, whereas 300 mm traffic lights are fixed at 500 cm above the road. In most cases, the traffic lights are not parallel with respect to the road, but they are slightly orientated toward it. In our case, the height of the traffic light (i.e., 190 cm) is not similar to the height specified by the standards, as it is quite difficult to handle such equipment in laboratory conditions. Therefore, the height and the orientation of the traffic light influence the power of the received signal and, in consequence, the SNR and the BER. Nevertheless, as the vehicular outdoor environment is highly dynamic and very unpredictable, an on vehicle VLC receiver will experience, in certain conditions, similar SNR levels, whereas, in other cases, the SNR could differ slightly. Even so, these results are still representative for traffic light to vehicle communication scenario, especially when the emitter–receiver distance is increasing. In such a case, the solid angle between the traffic light and the vehicle is decreasing and the influence of this height is reduced.

This article also intended to investigate and propose a VLC system that is able to work with different modulation techniques, coding techniques, and data rates adjustable in accordance with the existing context. Yet, the experimental results showed that, under the investigated conditions, rather similar BER results have been obtained for the three coding techniques, with the exception of the 50 kb/s data rate. Although different from what we expected, we consider that this is mainly due to the performances of the VLC receiver: (i) thus, the logarithmic transimpedance circuit is able to significantly reduce the influence of the ambient light, providing expanded dynamic range and preventing the photodiode saturation; (ii) the automatic gain control circuit is able to maintain a constant signal amplitude output, although its input signal varies significantly; (iii) the adaptive filters are optimally adjusted on the signal frequency; (iv) the decoding algorithm that is based on pulse width measurement allows for variations of the data signal period. Nevertheless, we still consider that the use of DSSS modulation is suitable for high priority data in low SNR conditions [35,36], the usage of OOK along with Manchester coding is suitable in medium SNR [5,24], whereas the Miller code could be an efficient solution in MIMO applications [33,34], based on previous experience and on the existing literature.

Another important contribution of this work is provided by one of the first experimental verifications of an automotive VLC system in snowfall conditions. These tests have showed and reconfirmed the highly disruptive effect of snowfall on outdoor VLC. For these particular tests, snowfall and heavy gust have increased the number of bit errors by up to 10,000 times. Nevertheless, even so, a raw BER of 10^−4^–10^−3^ has been achieved. Thus, these tests show that communication is still possible, whereas, with the help of error correcting codes, the BER can be significantly improved. Based on an intense experimental evaluation of the proposed VLC system, one can conclude that this work provided important evidence concerning the future use of the VLC technology in vehicular applications. Thus, such systems can provide BER results that can go down to 10^−7^ values. Nevertheless, in unfriendly working conditions, the BER can increase up to 10^−3^. However, the purpose of this work was to evaluate the physical layer of VLC applications. Accordingly, even if strong sunlight, snowfall, and blizzard depreciate BER performances, the use of error correcting codes can significantly improve these performances in order to provide reliable inter-vehicle connections.

Last but not least, the experimental tests have also confirmed that the proposed system is able to comply with the latency requirements. These tests have showed that the VLC prototype is able to provide latencies below 20 ms for messages of 400 bits. This aspect becomes very important, as it has been shown that, in high traffic densities, IEEE 802.11p 5.9 GHz DSRC systems can have difficulties in complying with this requirement. Furthermore, numerous other studies indicate the complementarity between the two wireless communication solutions [27], and they suggest that, for improved reliability, 5.9 GHz DSRC and VLC should be used together [17,27]. In such circumstances, the VLC technology can provide ultra-low latency communications between neighboring vehicle, while offloading the DSRC channel and contributing this way to improved performances in communication-based vehicle safety applications.

## 6. Conclusions

In the context of an increasing concern toward the development of enhanced solutions for traffic safety improvement, this article has addressed the issues that are related to the use of VLC systems in communication-based vehicle safety applications. A new VLC system has been introduced and its experimental evaluation has been presented. The system has been developed using a more problematic approach, based on a standard power VLC emitter and a wide-angle VLC receiver. Although this method is more suitable for automotive applications, the SNR is affected. The VLC system has been tested for variable communication ranges, under variable SNRs, and for different modulation and coding techniques.

This work has also investigated the opportunity of using adaptable modulation techniques, coding techniques, and data rates, depending on the existing context. Thus, this article has made a significant step toward a context adaptive VLC system that is capable of maximizing the performance in each environment.

Another important contribution of this article comes from the experimental evaluation of the system in variable conditions, especially in snowfall and blizzard conditions. These tests have showed the highly disruptive effect of these weather phenomena, and confirmed that the system is still able to maintain communication.

Future work on this project involves further enhancing the system’s robustness to noise and further increasing the communication distance. Additionally, this project involves the development of reconfigurable VLC architectures and context-aware VLC systems. These future works are meant to fully demonstrate and confirm the suitability of VLC systems for automotive applications.

## Figures and Tables

**Table 1:** Summary of the experimental parameters.

Parameter	Feature/Value
Emitter type	200 mm standard complying LED traffic light having an measured optical power of 190 μW/cm^2^ (at one meter)
Receiver type	±45°/±15° PIN photodiode-based receiver using a logarithmic transimpedance configuration
Emitter height	190 cm
Receiver height	85 cm
Noise power induced by the natural light	100–300 μW/cm^2^
Noise power induced by LED light	0–800 μW/cm^2^
Received signal power	190 μW/cm^2^–0.076μW/cm^2^
Emitter–receiver distance	1–50 m
Modulations, coding techniques and data rates	DSSS modulation with SIK coding: 3 kb/s
	OOK modulation with Manchester coding: 11, 24 and 50 kb/s
	OOK modulation with Miller coding: 11, 24 and 50 kb/s

**Table 2:** Summary of the experimental results for setup NO. 1.

Modulation	Coding	Data Rate (kb/s)	VLC Distance (m)	BER *
DSSS	SIK	3	18	<10^−6^
OOK	Manchester	11	18	<10^−6^
OOK	Miller	11	18	<10^−6^
OOK	Manchester	24	18	<10^−6^
OOK	Miller	24	18	<10^−6^
OOK	Manchester	50	18	<10^−6^
OOK	Miller	50	18	<10^−6^

* The BER < 10^−6^ has been achieved based on a minimum 10 data sets, each test of one-million bits.

**Table 3:** Summary of the experimental results for setup NO. 2.

Modulation	Coding	Data Rate (kb/s)	VLC Distance (m)	BER
DSSS	SIK	3	18	<10^−6^
OOK	Manchester	11	18	<10^−6^
OOK	Miller	11	18	<10^−6^
OOK	Manchester	24	18	<10^−6^
OOK	Miller	24	18	<10^−6^
OOK	Manchester	50	18	<10^−6^
OOK	Miller	50	18	<10^−6^

**Table 4:** Summary of the experimental results for setup NO. 3.

Modulation	Coding	Data Rate (kB/s)	VLC Distance (m)	BER *	Conditions
DSSS	SIK	3	40	<10^−6^	with HB perturbing LEDs
OOK	Manchester	11	40	<10^−6^	
OOK	Miller	11	40	<10^−6^	
OOK	Manchester	24	40	<10^−6^	
OOK	Miller	24	40	<10^−6^	
OOK	Manchester	50	40	5.71 × 10^−4^	with HB perturbing LEDs
OOK	Miller	50	40	2.23 × 10^−3^	
OOK	Manchester	50	40	<10^−6^	without HB perturbing LEDs
OOK	Miller	50	40	1.7/10^−6^	

* The bit error ration (BER) < 10^−6^ has been achieved based on minimum 10 data sets, each test of one million bits.

**Table 5:** Summary of the weather conditions during the outdoor tests.

Weather Parameter	Numeric Value Range
Temperature [°C]	−0.5–−2.3
Relative humidity [%]	94–96
Snowfall amount [cm]	1.5
Shortwave radiation [W/m^2^]	45–76
Wind speed [km/h]	60–63
Wind gust [km/h]	58–64

**Table 6:** Summary of the VLC system configuration during the outdoor tests in snowfall conditions.

VLC System Feature	Configuration
Modulation	OOK
Coding	Manchester
Data rates	11 kb/s; 24 kb/s; 50 kb/s
VLC distance	50 m
VLC receiver FOV	±15°

**Table 7:** Summary of the experimental results in snowfall conditions.

Conditions	BER
Low parasitic lighting	<10^−6^ *
Snowfall conditions	4 × 10^−5^
Snowfall and heavy gust conditions	Up to 10^−3^

* The BER < 10^−6^ is established based on data sets up to 10 million bits.

**Table 8:** Summary of the experimental results showing the VLC prototype latency performances for different modulation techniques, coding techniques and data rates.

Modulation	Coding	Data Rate (kb/s)	VLC Distance (m)	Message Length (bits)	Latency (ms)
DSSS	SIK	3	18	400	118
OOK	Manchester	11	18	400	32.8
OOK	Miller	11	18	400	32.8
OOK	Manchester	24	18	400	15.6
OOK	Miller	24	18	400	15.6
OOK	Manchester	50	18	400	8.12
OOK	Miller	50	18	400	8.12
